# The inflammation-pyroptosis axis links local and systemic biomarker profiles to active alveolar bone microstructural deterioration

**DOI:** 10.3389/fphys.2026.1814322

**Published:** 2026-05-13

**Authors:** Yawen Cui, Yifan Xuan, Leyi Wang, Jinxin Yang, WenTing Song, Zongxiang Liu

**Affiliations:** 1 Xuzhou Medical University School of Stomatology, Xuzhou, China; 2 The Affiliated Stomatological Hospital of Xuzhou Medical University, Xuzhou, China

**Keywords:** alveolar bone destruction, biomarkers, oxidative stress, periodontitis, pyroptosis

## Abstract

**Introduction:**

This study aimed to identify biomarkers in saliva and serum that are highly correlated with the degree of bone destruction, and to further explore potential mechanisms, thereby providing evidence for accurate diagnosis and treatment of periodontitis.

**Methods:**

A periodontitis model was established using maxillary molar ligation. Bone microstructural parameters were measured by Micro-CT. A multiplex liquid chip assay measured 21 biomarkers in saliva and serum, including inflammatory, oxidative stress, matrix-degrading, and bone remodeling factors. XGBoost was applied to select the key biomarkers, and we confirmed the findings with multiple linear regression and correlation tests. Histological assessments were performed to validate biomarker reliability and to investigate potential mechanisms.

**Results:**

Micro-CT showed significant differences in BV/TV, Tb.Sp, and CEJ–ABC among groups (P < 0.05); XGBoost identified monocyte chemoattractant protein-1 (MCP-1), tumor necrosis factor-alpha (TNF-α), and caspase-1 (CASP-1) as the main contributors. In saliva, these markers had fairly even contributions (27.7%, 34.4%, 18.4%); in serum, MCP-1 was the strongest (67.7%), followed by TNF-α (20.5%) and CASP-1 (8.4%). These key biomarkers correlated positively with Tb.Sp and CEJ-ABC, and they colocalized with gasdermin D (GSDMD) in areas of severe damage, which suggests a role in pyroptosis-related bone destruction.

**Discussion:**

Inflammatory signals in saliva and serum can reflect active destruction of alveolar bone micro-structure in periodontitis. The joint increase in key markers and their close location with GSDMD suggest that the mechanism may involve the pyroptosis pathway. These findings point to possible targets for treating periodontal bone loss and for managing the links between local bone damage and systemic diseases.

## 1 Introduction

Periodontitis is a chronic inflammatory disease characterized by progressive destruction of the periodontal supporting tissues, particularly pathological resorption of the alveolar bone. This process not only impairs, and may even lead to loss of, masticatory function, but also forms a bidirectional regulatory network with systemic diseases such as cardiovascular disease, diabetes mellitus, and malignant tumors (Salari and Alavi, 2021). Therefore, elucidating the molecular mechanisms underlying the progression of alveolar bone resorption in periodontitis and identifying the key drivers of its sustained aggravation, as well as screening sensitive biomarkers that accurately reflect the course of bone destruction, are of important clinical value for achieving precision diagnosis and treatment of periodontitis. The concept that alveolar bone resorption results from the synergistic action of multiple destructive factors has been widely accepted. The process may initiate with inflammatory mediator–driven osteoclastogenesis: periodontal pathogens and their products act as initiating stimuli, triggering innate and adaptive immune responses, leading to extensive infiltration of inflammatory cells and release of pro-inflammatory cytokines, including tumor necrosis factor-alpha (TNF-α), interleukin-1 beta (IL-1β), interleukin-6 (IL-6), interleukin-17 (IL-17), and chemokines such as monocyte chemoattractant protein-1 (MCP-1) (AlQranei et al., 2021; Cafferata et al., 2021; Usui et al., 2021). These mediators increase the receptor activator of nuclear factor-κB ligand/osteoprotegerin (RANKL/OPG) ratio within osteoclast differentiation and activation pathways, thereby promoting the differentiation of bone marrow–derived precursor cells into osteoclasts and enhancing osteoclastic resorptive activity, ultimately resulting in pathological alveolar bone resorption. Under persistent chronic inflammatory stimulation, activated neutrophils and macrophages overproduce reactive oxygen species (ROS) through the NADPH oxidase complex and electron leakage from the mitochondrial respiratory chain, causing pronounced oxidative stress injury (Bassani et al., 2023; Wang et al., 2023). Excessive ROS induces periodontal tissue cell damage via lipid peroxidation, protein carbonylation, and DNA strand breaks (Chen et al., 2023; Shang et al., 2023), and also acts as a signaling molecule to activate the NLRP3 inflammasome, driving caspase-1–dependent pyroptosis and the release of mature IL-1β and IL-18. This amplifies the local inflammatory cascade, reinforces the inflammatory microenvironment, and increases osteoclast sensitivity to RANKL (Zhu et al., 2020; Song et al., 2023). Meanwhile, inflammatory and oxidative stress signals stimulate periodontal ligament fibroblasts and inflammatory cells to release large amounts of matrix metalloproteinases (MMPs) and tissue-degrading enzymes, such as MMP-8 and CTSK (Kondo et al., 2022; Luchian et al., 2022), which extensively degrade extracellular matrix components including collagen fibers. This further leads to loss of periodontal ligament attachment and creates physical conditions conducive to osteoclast attachment to bone surfaces and resorptive activity. Ultimately, these destructive factors collectively cause dysregulation of tissue metabolic factors such as LDH, VEGF-A, PECAM-1, and ALP (Sadek et al., 2023), suppress osteoblast activity, and shift bone remodeling balance further toward pathological degradation. Early diagnosis and interruption of alveolar bone resorption are important strategies for treating periodontitis; however, clinical diagnosis mainly relies on imaging and clinical examinations, which are limited by substantial lag, high subjectivity, and difficulty in accurately quantifying early microstructural changes. Local minocycline hydrochloride administration and photodynamic non-surgical therapy can effectively control local inflammation and plaque, but their ability to promote alveolar bone repair remains limited. Therefore, developing molecular biomarker–based techniques that can accurately predict the degree of bone resorption, guide individualized drug intervention, and enable chairside quantitative testing (POCT) is warranted.

Biomarker research is currently a major focus, yet most studies have concentrated on inflammation-related factors or on coarse discrimination between health and disease states (Arias-Bujanda et al., 2020; Kawamoto et al., 2020), while relatively little attention has been paid to direct associations between biomarkers and changes in bone microarchitecture. Many studies have screened biomarkers and built predictive models based on a single sample source (Huang et al., 2020; Kim et al., 2020), lacking comparisons between local and systemic samples and dynamic evaluation across disease grades. This, to some extent, reduces the ability of predictive models to reflect the bone resorption process and limits validation of key factors involved in the interaction between periodontitis and related systemic diseases. In addition, many biomarkers have insufficient specificity and are readily influenced by genetic background, lifestyle, and environmental factors, resulting in poor stability and consistency of findings (Belstrøm et al., 2017). Methodologically, existing analyses often rely on conventional statistics and retrospective designs, which tend to interpret complex multidimensional data in a linear manner and may overlook the contribution of early key determinants.

In the present study, we aimed to identify which biofluid biomarkers most closely reflect the severity of active alveolar bone microstructural deterioration in periodontitis. To address this question, we used a standardized ligature-induced rat model and simultaneously profiled 21 candidate biomarkers in saliva and serum. By integrating micro-CT-based bone grading, XGBoost feature prioritization, correlation and regression analyses, and tissue-level histological validation, we sought to identify a multi-biomarker panel associated with progressive bone destruction. Therefore, the novelty of this study lies in linking local and systemic biofluid signals with quantitative alveolar bone microarchitecture, rather than in proposing any single biomarker as entirely novel in periodontitis.

## 2 Materials and methods

**Figure 1:**
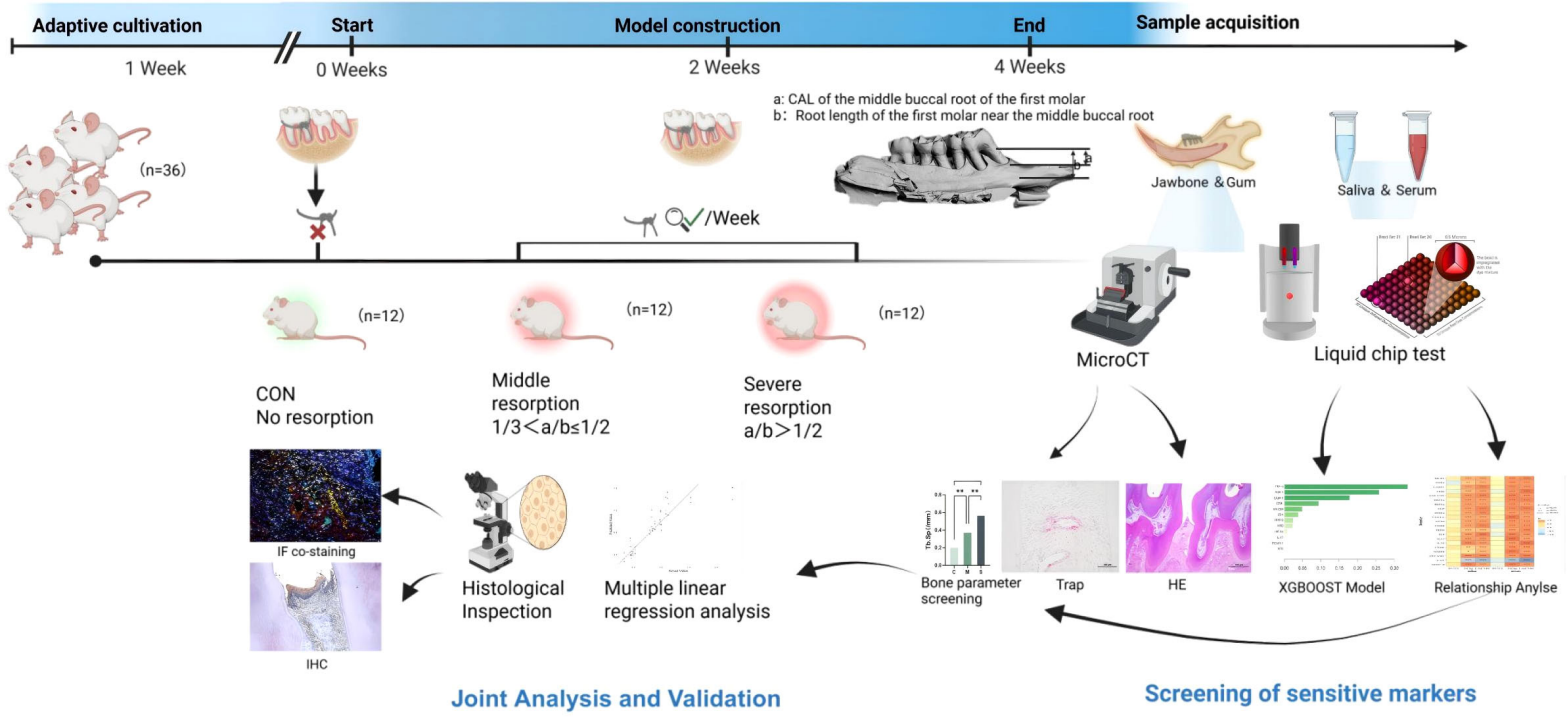
Schematic diagram of the experimental workflow.

According to the experimental workflow (**Figure 1**), a quantitative grading system for alveolar bone resorption was established based on micro-CT–quantified bone microarchitectural parameters, including bone volume fraction (BV/TV), trabecular separation (Tb.Sp)and CEJ–ABC. A multiplex liquid chip assay was used to simultaneously measure 21 candidate biomarkers in saliva and serum, covering pathways related to inflammation, oxidative stress/pyroptosis, matrix degradation, tissue remodeling, and bone metabolism. Fordata analysis, descriptive statistics were first performed for all measured values, including between-group differential expression and relative expression levels; XGBoost was then applied to model the biomarkers and rank feature importance. Given the small sample size and the tabular nature of the dataset, XGBoost was used primarily as an exploratory and interpretable feature-prioritization tool rather than as a standalone predictive model for broad generalization. Its role in this study was to narrow the candidate biomarker set and guide subsequent statistical validation. On this basis, correlation analyses and multivariate regression models were further conducted to quantify, from a linear perspective, the strength and direction of associations between biomarkers and bone parameters. Finally, immunohistochemistry and multiplex immunofluorescence were used to validate and localize biomarker expression at the tissue level in alveolar bone.

### 2.1 Reagents and equipment

TRAP staining kit (Solarbio, China); protease inhibitor cocktail (Sigma-Aldrich, USA); antigen retrieval solution, pH 6.0 (Sigma-Aldrich, USA); DAB chromogenic kit (Zhongshan Bio, China); 5% bovine serum albumin (BSA) (Sigma-Aldrich, USA); hematoxylin staining solution (Kaiji, China); Luminex multiplex assay kit (Luminex, USA); anti-GSDMD (1:200, Cat# ab219800, Abcam, UK); anti-IL-1β (1:100, Cat# 503501, BioLegend, USA); anti-TNF-α (1:100, Cat# 506101, BioLegend); anti-MCP-1 (1:200, Cat# 505901, BioLegend); Alexa Fluor 488/594/647/555 (1:500, Cat# GB25303/GB28404/GB27301/GB26301,Servicebio, China); sodium pentobarbital (Sigma-Aldrich, USA).

Micro-CT scanner (Scanco, Switzerland); paraffin embedding station (Junjie Electronics, China); optical microscope imaging system (Leica, Germany); Luminex 200 multiplex analyzer (Luminex, USA); inverted fluorescence microscope (Olympus, Japan).

### 2.2 Experimental animals

Forty specific pathogen-free (SPF) healthy Sprague–Dawley (SD) rats (male), 8 weeks old, weighing 200 ± 20 g, were used. The rats were housed in clean cages under controlled conditions (25 °C; 60%–70% relative humidity) with a 12h light/12h dark cycle. All animals received the same chow and had free access to food and water; they were housed separately by cages. After 1 week of acclimatization, rats were assigned to groups using a random number table. (Approval: No. 202502T017).

Sample size was estimated using G*Power (v3.1.9.7) based on pilot CEJ–ABC data (one-way ANOVA, α = 0.05, power = 0.80,f = 0.42), indicating a minimum of 10 animals per group. To ensure adequate statistical power, 12 rats were allocated to each group; considering an approximately 20% attrition rate, a total of 40 rats were enrolled.

After 1 week of acclimatization, rats were randomly assigned to three groups: severe periodontitis (S group, n = 14), moderate periodontitis (M group, n = 14), and normal control (C group, n = 12). Different ligature durations were selected to generate distinguishable stages of alveolar bone destruction under otherwise controlled experimental conditions, thereby facilitating subsequent micro-CT-based grading of moderate versus severe periodontitis. Experimental periodontitis was induced by ligature placement around the maxillary first and second molars. In the S group, ligatures were maintained for 4 weeks; in the M group, for 2 weeks; controls received transient ligature placement without sustained retention. At the end of week 4, rats were euthanized, and alveolar bone loss around the mesiobuccal root of the maxillary first molar was measured by micro-CT and expressed as the percentage of resorption length relative to root length. Rats meeting the predefined micro-CT criteria for bone loss severity were included in subsequent analyses. The thresholds for moderate periodontitis (33% < alveolar bone resorption ≤ 50% of root length) and severe periodontitis (alveolar bone resorption > 50% of root length) were specified *a priori* with reference to previously reported micro-CT-based grading criteria (Fernandes et al., 2024), rather than being derived from the distribution of the current dataset.

### 2.3 Selection and classification of biomarkers

Based on a literature review, bone destruction– and inflammation-related factors closely associated with periodontitis, bone metabolism, and related systemic diseases (e.g., diabetic nephropathy and non-alcoholic fatty liver disease) were identified, and 21 candidate molecules that can be stably quantified on the Luminex platform were selected, covering key driving processes from soft tissue injury to alveolar bone resorption. (**Supplementary Figures**). These molecules were categorized into four groups according to their predominant functions, which facilitates interpretation of the biomarker profile while also maintaining controllability of marker number and assay cost for subsequent translation to multi-biomarker POCT platforms.

### 2.4 Sample collection and storage

At the end of week 4 after modeling, rats were fasted for 2 h. To minimize the influence of circadian rhythms on biomarker levels, serum and saliva samples were collected between 9:00 and 11:00 a.m. Rats were anesthetized by intraperitoneal injection of sodium pentobarbital (40 mg/kg). Whole blood (5 mL) was obtained by cardiac puncture at the point of maximal apical impulse and collected into yellow serum-separator (procoagulant) tubes. After standing at room temperature for 30 min to allow clotting, samples were centrifuged at 4 °C and 3,000 × g for 15 min. The supernatant serum was aliquoted (250 μL per tube) and stored at −80 °C until analysis. Pilocarpine (1 mg/kg, intraperitoneal) was then administered to stimulate salivation. Rats were placed on their side with the head slightly lowered. Whole saliva was collected using a sterile pipette and quickly moved (on ice) to 1.5-mL low-binding microcentrifuge tubes. The samples were centrifuged at 4 °C and 15,000 × g for 15 minutes. We took 50 μL of the clear supernatant, added protease inhibitor cocktail to reach a final concentration of 1%, mixed it well, and stored the samples at −80 °C. Samples with visible blood or too little volume were not used for further analysis.

After collecting serum and saliva, the rats were euthanized with an overdose of sodium pentobarbital. The maxilla was dissected intact, and hemimaxillary specimens containing the first and second molars and surrounding intact periodontal tissues were harvested. All specimens were fixed in 4% paraformaldehyde (prepared in PBS, pH 7.4) at 4 °C. For histological analysis, specimens were fixed for 24 h and then decalcified in 10% EDTA solution (pH 7.4) at 4 °C, with the decalcification solution replaced every 2–3 days for approximately 20 days; completion of decalcification was confirmed using the needle-probing method. After decalcification, specimens were rinsed under running tap water for 6 h, post-fixed again in 4% paraformaldehyde for 4 h, thoroughly washed for 24 h, and then transferred to 75% ethanol and stored at 4 °C until paraffin embedding. The contralateral hemimaxilla for micro-CT analysis was fixed in 4% paraformaldehyde for 48 h, transferred to 70% ethanol, and stored long-term at 4 °C.

### 2.5 Clinical and histological evaluation

Gingival index (GI), and probing depth (PD) were assessed by same investigator. Histological evaluation was performed using hematoxylin–eosin (H&E) staining to assess tissue architecture and inflammatory infiltration. Tartrate-resistant acid phosphatase (TRAP) staining was performed to evaluate osteoclast presence. TRAP-positive multinucleated cells were quantified within standardized regions of interest.

### 2.6 Micro-CT analysis of alveolar bone

Bone morphology and changes in microstructural parameters were evaluated by micro-CT scanning and three-dimensional reconstruction. The maxillae were scanned using a high-resolution micro-CT system (50 kV, 200 μA; voxel size 10 μm). Three-dimensional reconstruction was performed with analysis software (Mimics Medical 21.0), after which the distance from the alveolar crest to the cemento-enamel junction at the midpoint of the buccal roots of M1 and M2 (along the long axis of the root) was measured and defined as CEJ–ABC. Samples meeting the grading threshold of CEJ–ABC/root length ≥ 50% were identified based on this measurement. The region of interest (ROI) was defined as the buccal alveolar bone in the interdental area between M1 and M2, extending 1.5 mm apically from a point 0.5 mm below the furcation, with a thickness of 100 slices (1 mm). Using a fixed grayscale threshold applied uniformly across all groups, quantitative analyses were performed for BV/TV, Tb.Th, Tb.N and Tb.Sp in the molar region.

### 2.7 Luminex assay and quantification

A multiplex immunoassay platform based on Luminex bead-based technology was used to measure target biomarker levels in saliva and serum. Prior to testing, the instrument was calibrated and performance-verified using the calibration kit. Fifty microliters of each serum and saliva sample were used to measure concentrations. After establishing standard curves, the Luminex xPONENT 4.2 software was used to input the MFI values of each sample into the fitted standard-curve equation (5-parameter logistic inverse function) to back-calculate concentrations and output absolute concentrations (pg/mL) for quantitative analysis. Biomarker levels were normalized to the control group for relative expression analysis.

### 2.8 Non-linear variable importance analysis

To evaluate the discriminatory value of biomarkers across different grades of bone resorption, separate XGBoost models were constructed for saliva and serum. Given the relatively small sample size and the tabular structure of the dataset, XGBoost was used primarily as an exploratory and interpretable feature-prioritization tool rather than as a standalone predictive model for broad generalization. Its role in this study was to narrow the candidate biomarker set and guide subsequent statistical validation.

All samples were randomly split into training and test sets at a 7:3 ratio while maintaining the same group proportions. The same parameter settings were applied to the saliva and serum models to facilitate comparison of feature-ranking patterns between the two biofluids. The model was implemented in R (version 4.5.2) with the following parameters:

- nrounds = 100.
- max_depth = 5.
- learning_rate = 0.3.
- objective = “multi:softprob”.
- random seed fixed for reproducibility.

To reduce the risk of overfitting under small-sample conditions, stratified five-fold cross-validation was applied during internal model evaluation. The resulting feature-importance rankings were interpreted together with correlation analysis, multiple linear regression, and tissue-level validation, rather than being considered definitive on the basis of the machine-learning output alone.

### 2.9 Correlation and multiple linear regression analyses

To further check the link between biomarker levels and the amount of alveolar bone destruction, and to confirm the XGBoost feature ranking, we used the concentrations of the 21 biomarkers as independent variables and the main Micro-CT bone parameters as dependent variables. Correlation coefficients were calculated to assess the strength and significance of these associations, and the results were showed in a heatmap.

Three types of regression models were constructed: a salivary biomarker model, a serum biomarker model, and a combined salivary–serum biomarker model. The key biomarkers served as independent variables, while the main Micro-CT bone parameters were the dependent variables. Multiple linear regression was fitted using the lm function. We assessed the model’s accuracy with two metrics: the coefficient of determination (R^2^) and mean squared error (MSE). We also compared a model with the “Top 3” biomarkers against one using only the “Top 1” biomarker to quantify the predictive advantage of the combined panel.

### 2.10 Immunohistochemistry and multiplex immunofluorescence

Immunohistochemistry was used to show the location of MCP-1, TNF-α, and CASP-1 expression in periodontal tissues. Sections were incubated with primary antibodies overnight at 4 °C, followed by secondary antibody incubation and DAB visualization.

Multiplex immunofluorescence co-staining was done to assess spatial colocalization between key biomarkers and the pyroptosis executor GSDMD. We used sequential staining to prevent cross-reactivity between antibodies. Images were taken with a confocal microscope, and we measured fluorescence intensity and colocalization coefficients.

### 2.11 Statistical analysis

Statistical analyses were performed using GraphPad Prism (version 10.1.2) and R (version 4.3.2). During data preprocessing, values more than three standard deviations from the mean were flagged as outliers according to a prespecified criterion and excluded from the corresponding biomarker analysis. Before comparing groups, normality and homogeneity of variances were assessed using the Shapiro–Wilk test and Levene’s test, respectively. For variables that met normality and homoscedasticity assumptions, one-way analysis of variance (ANOVA) was used; otherwise, the Kruskal–Wallis rank-sum test was applied. When the overall test showed a significant difference, Tukey’s *post hoc* test was used for normally distributed data and Dunn’s test for non-normal data. For correlation analyses, Pearson’s correlation was used for normally distributed variables and Spearman’s rank correlation for others. The performance of multiple linear regression models was assessed using the coefficient of determination (R^2^) and mean squared error (MSE). Statistical significance was defined as P < 0.05.

## 3 Results

### 3.1 Establishment and validation of the rat periodontitis model

**Figure 2:**
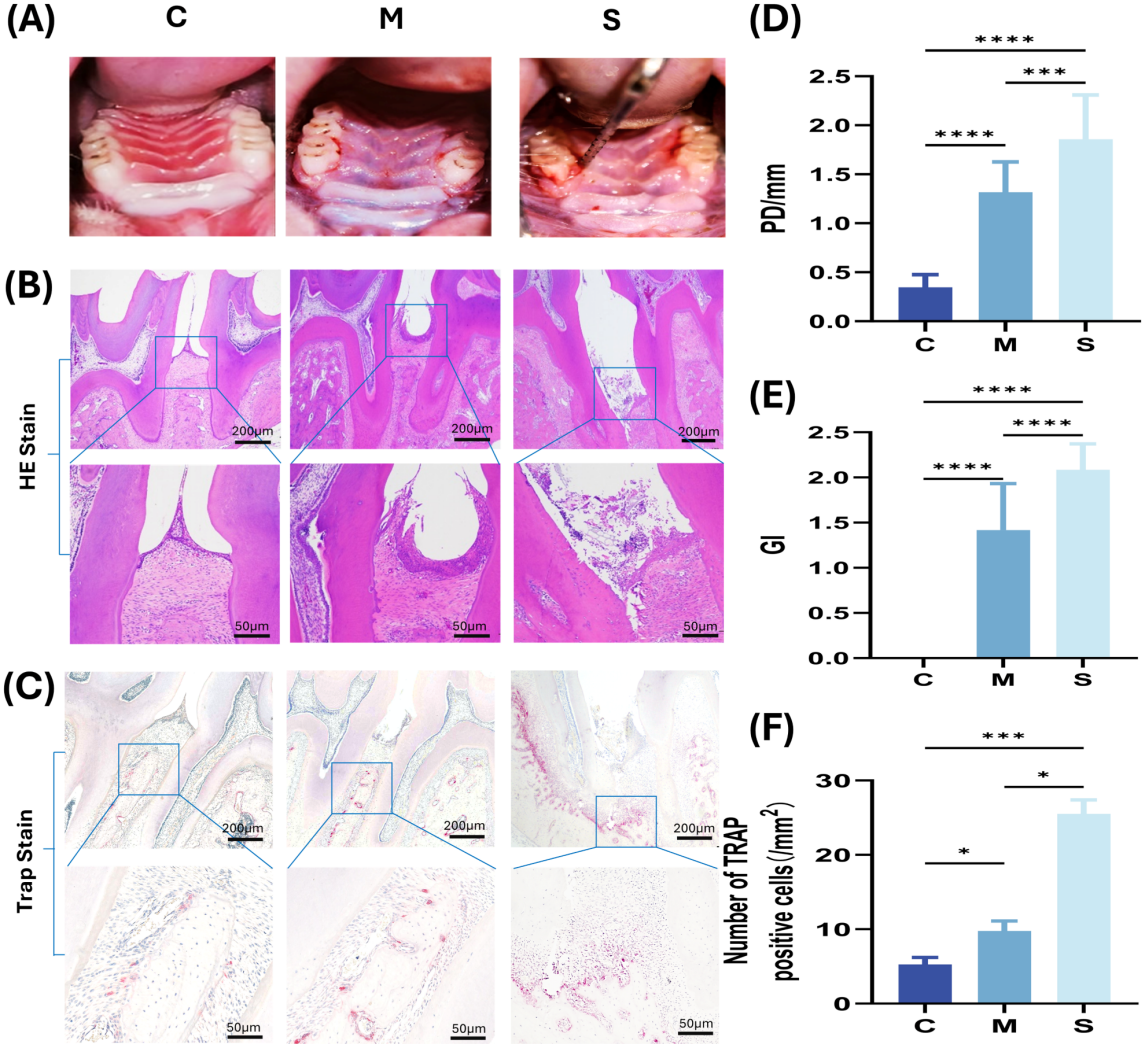
Establishment and validation of the rat periodontitis model **(A)** Intraoral photographs of rats. **(B)** H&E staining (4× and 20×). **(C)** TRAP staining (4× and 20×). **(D, E)** Between-group comparisons of GI and PD. **(F)** Quantification of TRAP-positive cells per 1 mm^2^. *P < 0.05; **P < 0.01; ***P < 0.001; ****P < 0.0001.

A total of 28 rats were initially modeled. According to the predefined micro-CT-based grading criteria for alveolar bone resorption, animals that did not meet the *a priori* inclusion thresholds were excluded, and 12 rats per group were ultimately retained for subsequent experiments. Gross observation showed that the gingiva in the control group (C) was pink, with a thin gingival margin closely adapted to the tooth surface and a firm texture, whereas the gingiva in the M and S groups was hyperemic and swollen with a soft texture; swelling was more pronounced in the S group (**Figure 2A**). As shown by the gingival index (GI) (**Figure 2E**), no redness, swelling, or bleeding was observed in the C group, while both the M and S groups exhibited bleeding on probing, with significant differences compared with the C group (P < 0.0001); a significant difference was also observed between the M and S groups (P < 0.05). Probing depth (PD) results (**Figure 2D**) showed shallow periodontal pockets in the C group; compared with the C group, PD was markedly increased in both the M and S groups (P < 0.001), and a significant difference was also detected between the M and S groups (P < 0.05). H&E staining revealed intact periodontal tissue architecture in the C group, with a clearly defined keratinized layer and no inflammatory infiltration in the lamina propria; in contrast, epithelial continuity was disrupted in the M and S groups, the gingival papillae were poorly defined, inflammatory cells were widely infiltrated, and the junctional epithelium migrated apically (**Figure 2B**). TRAP staining showed only a few TRAP-positive osteoclasts on the alveolar bone surface in the C group; in the M group, osteoclast numbers were markedly increased, with enlarged cell bodies and clustering along the margins of resorption lacunae; in the S group, dense clusters of TRAP-positive cells were observed, with further enlargement and typical coalescence of resorption lacunae forming a “bone erosion front-like” structure. Quantitative analysis of TRAP-positive multinucleated cells also showed statistically significant differences among groups (all P < 0.05) (**Figures 2C, F**).

### 3.2 Bone reconstruction and selection of microstructural bone parameters

**Figure 3:**
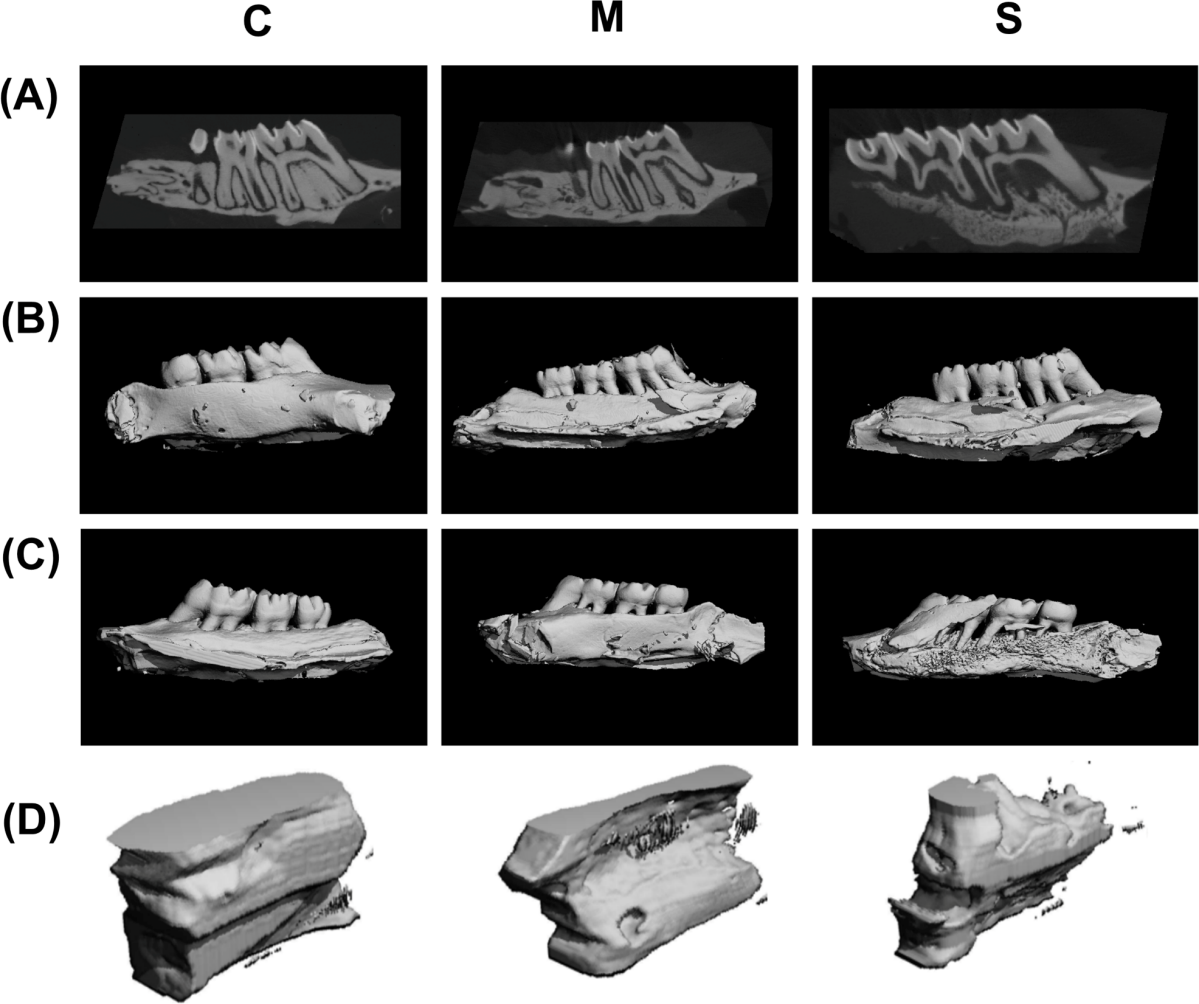
Three-dimensional micro-CT reconstruction of alveolar bone destruction in the rat periodontitis model. **(A)** Transverse view. **(B)** Palatal view. **(C)** Buccal view. **(D)** Schematic of bone microarchitecture in the ROI.

Observation of the three-dimensional reconstructions from the transverse, palatal, and buccal views showed that the C group exhibited an intact overall contour of the alveolar crest with continuous cortical bone. In the M group, the alveolar crest contour was markedly irregular, bone height was significantly reduced, and bone density was slightly lower than that in the C group. In the S group, the alveolar crest contour was severely disrupted, bone height decreased to the apical level, and bone density appeared sparse. The extent of destruction was consistent with the clinical and histopathological findings and matched the group classifications (**Figure 3**).

**Figure 4:**
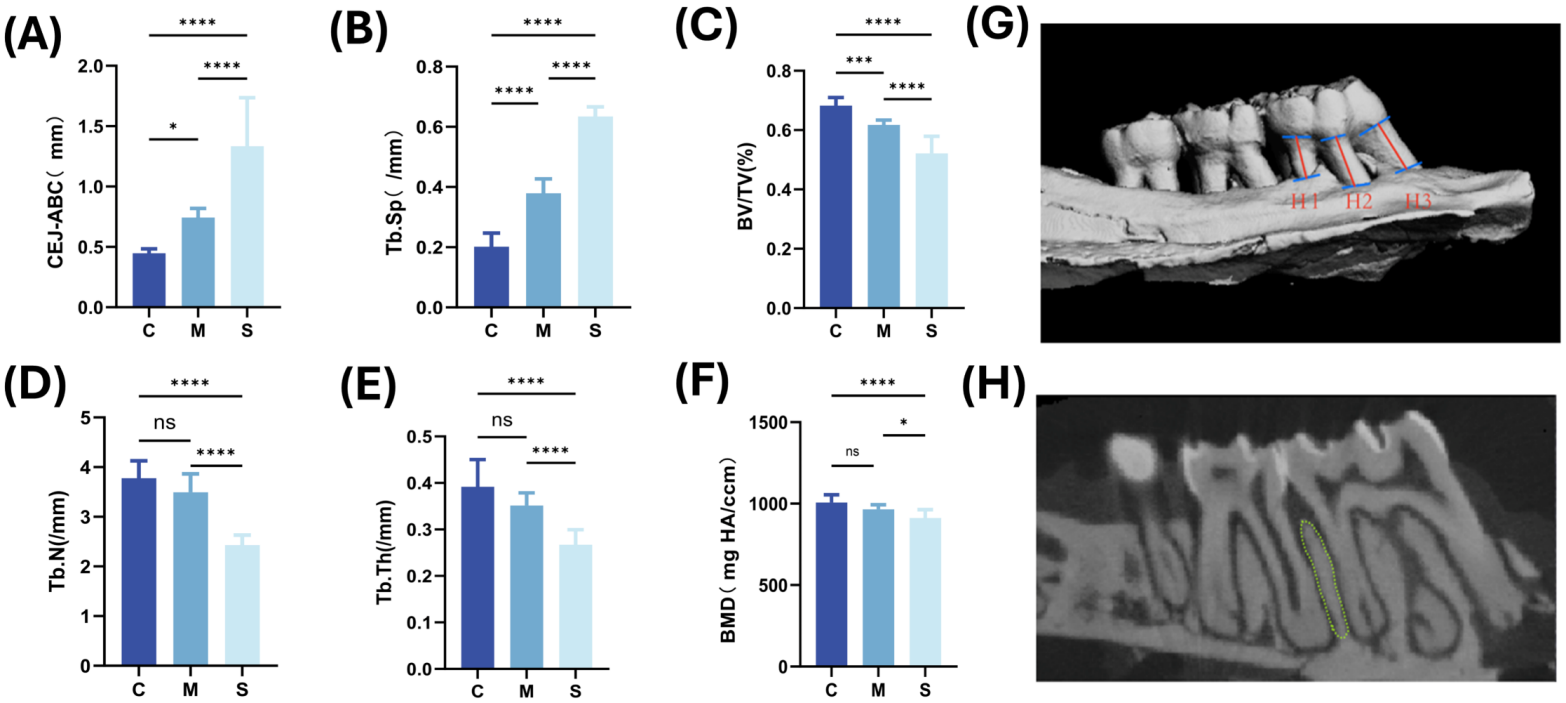
Differential expression of bone parameters and definition of the ROI **(A–F)** Differential analyses of CEJ–ABC, BV/TV (bone volume fraction), Tb.Sp (trabecular separation), Tb.N (trabecular number), Tb.Th (trabecular thickness), and BMD (bone mineral density). **(G)** For each root of the first molar, the distance from the midpoint of the cemento-enamel junction along the long axis of the root to the alveolar crest was measured and denoted as H1, H2, and H3; the mean value was defined as CEJ–ABC. **(H)** Schematic illustration of bone microarchitecture showing the selected ROI. *P < 0.05; **P < 0.01; ***P < 0.001; ****P < 0.0001.

After quantitative analysis of the ROI (**Figures 4G, H**), CEJ–ABC and Tb.Sp increased significantly across groups, whereas BV/TV, Tb.N, Tb.Th, and BMD showed decreasing trends (**Figures 4A–F**). Compared with the C group, BV/TV was significantly reduced in the M group (P < 0.05), whereas Tb.Th, Tb.N, and BMD exhibited downward trends without reaching statistical significance (P > 0.05), indicating that these three parameters changed less markedly in the early stage of bone resorption. In contrast, Tb.Sp and CEJ–ABC were significantly increased (P < 0.001). All parameters differed significantly between the M and S groups. Based on these findings, BV/TV, CEJ–ABC, and Tb.Sp were selected as key parameters that more accurately reflect bone mass loss and microstructural destruction for establishing biomarker-based predictive models.

### 3.3 Biomarker measurement and quantification

**Figure 5:**
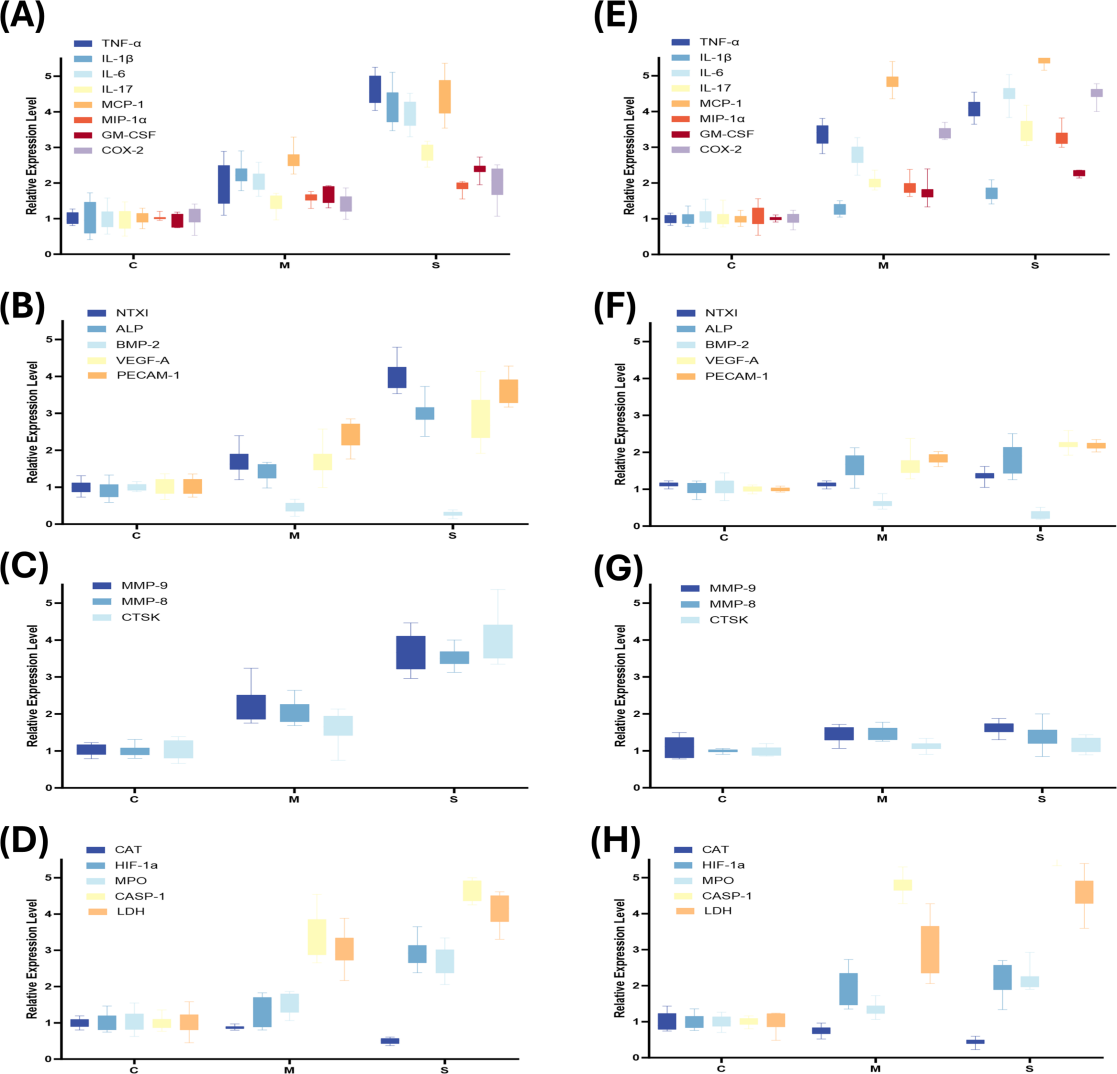
Box plots of relative expression levels of biomarkers across groups **(A–D)** Relative expression levels of four functional categories of biomarkers in saliva. **(E–H)** Relative expression levels of four functional categories of biomarkers in serum. *P < 0.05; **P < 0.01; ***P < 0.001; ****P < 0.0001.

After normalization of the 21 biomarkers measured by Luminex to the C group, most biomarkers in both the M and S groups were upregulated to varying degrees in saliva and serum, showing clear differences in biofluid source and magnitude of expression (**Figure 5**). As a local microenvironmental sample, saliva exhibited increases in most measured biomarkers, suggesting broad activation of local inflammation and tissue metabolism. In contrast, biomarkers that were markedly elevated in serum were relatively concentrated, mainly including MCP-1, IL-1β, TNF-α, CASP-1, and LDH—limited inflammatory mediators and oxidative stress–related molecules—indicating that the systemic amplification effect of serum biomarkers is more intensely manifested in the dimensions of inflammation and oxidative stress. Notably, MCP-1, TNF-α, CASP-1, LDH, IL-6, and IL-1β showed the most prominent increases in both saliva and serum and displayed a graded increase with the severity of bone resorption (P < 0.001), suggesting that they may serve as candidate core biomarkers for grading bone destruction and linking local and systemic status.

### 3.4 XGBoost model construction and identification of key biomarkers

**Figure 6:**
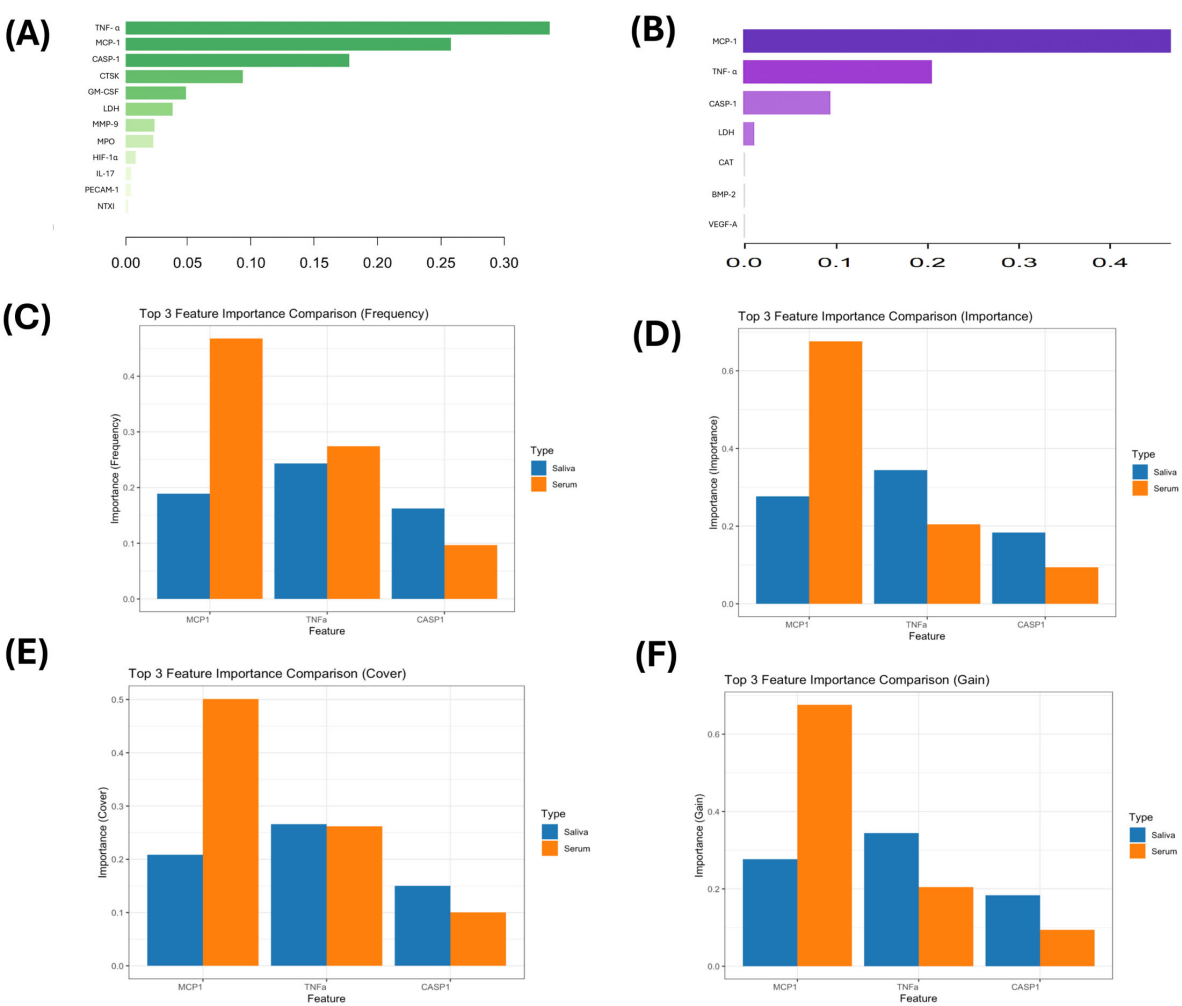
Feature-importance results and multidimensional comparison of key biomarkers **(A)**Feature-importance scores of biomarkers in saliva. **(B)** Feature-importance scores of biomarkers in serum. **(C–F)** Comparison of TNF-α, MCP-1, and CASP-1 across four metrics: frequency, importance, cover, and gain.

After ranking the 21 cytokines by XGBoost’s built-in gain metric, five key factors were identified in saliva (TNF-α, MCP-1, CASP-1, CTSK, and GM-CSF) and three key factors were identified in serum (**Figures 6A, B**). Notably, the overlapping factors TNF-α, MCP-1, and CASP-1 exhibited distinct contribution patterns between the two biofluids. Comprehensive evaluation using gain, cover, frequency, and importance metrics (**Figures 6C–F**) showed that, in the salivary model, the contributions of the three core inflammatory factors were relatively balanced and stable, ranked as TNF-α, MCP-1, and CASP-1, with high consistency across metrics. In the serum model, MCP-1 contributed up to 67.6% and played the main role in predictions, while other factors had much smaller contributions. This suggests that serum biomarker predictions in this study may depend mostly on one strong signal. Besides the core inflammatory axis, biomarkers related to metabolism and tissue injury (e.g., LDH, CAT, and CTSK) showed different patterns in saliva compared to serum, indicating a potential advantage of saliva in capturing composite signals of local inflammation and systemic injury.

### 3.5 Correlation analysis between biomarker levels and microstructural bone parameters

**Figure 7:**
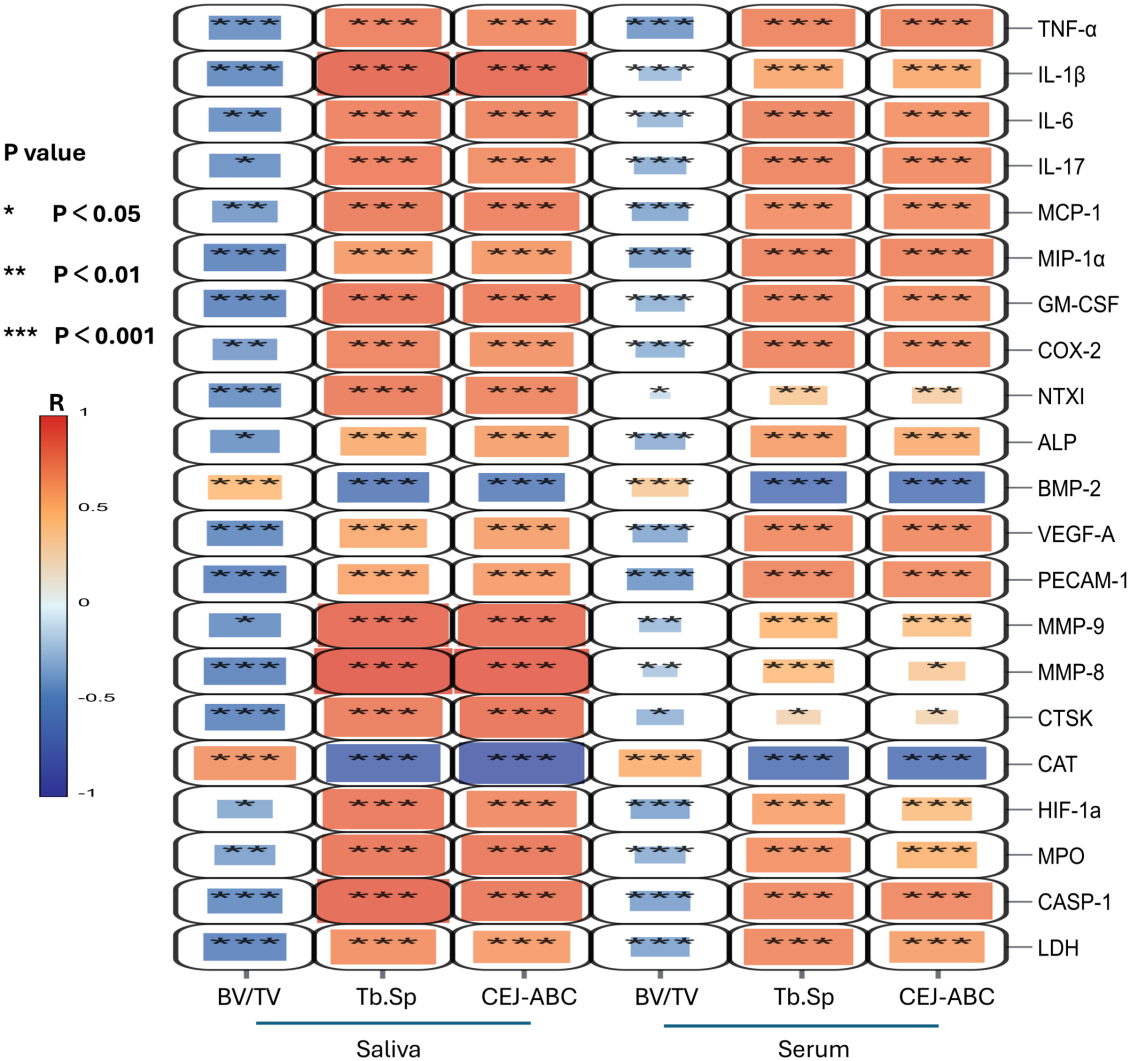
Correlation heatmap of biomarkers and bone parameters in saliva and serum. Spearman correlation heatmap showing associations between 21 biomarkers and bone-related parameters (BV/TV, Tb.Sp, and CEJ–ABC) in saliva and serum. *P < 0.05, **P < 0.01, ***P < 0.001. Color scale for R values increases from blue (negative correlation) to red (positive correlation).

The Shapiro–Wilk normality test showed that, in saliva, eight indicators (MIP1a, HIF1a, BMP2, NTXI, COX2, IL17, MMP9, and PECAM-1) were non-normally distributed (P < 0.05), whereas in serum, all indicators except CAT (20/21) were non-normally distributed. Spearman correlation analysis demonstrated (**Figure 7**) that inflammatory mediators and tissue degradation–related factors, including TNF-α, IL-1β, IL-17, MMP-8, MMP-9, and CTSK, were highly positively correlated with Tb.Sp and CEJ–ABC (R>0.8, P < 0.001), while their associations with BV/TV were mostly weakly negative(R<0.55, P > 0.05). Oxidative stress and pyroptosis-related molecules, such as CASP-1, MPO, and HIF-1α, showed correlation patterns that fell between these two bone parameter categories, suggesting that they may play a unique regulatory role in inflammation-induced bone remodeling. The high-correlation distribution in serum is primarily concentrated among inflammatory cytokines, whereas the correlations of tissue degradation and metabolism markers with bone parameters are weaker in serum than those observed in saliva. Focusing on the key factors identified by XGBoost (MCP-1, TNF-α, and CASP-1), correlation analysis showed that all three were significantly positively correlated with Tb.Sp and CEJ–ABC in both saliva and serum, with correlation coefficients (R > 0.75, P < 0.001) (**Figure 7**). These results are consistent with the micro-CT findings showing a stepwise increase in bone resorption severity across disease grades and align with the feature-importance ranking produced by the XGBoost model, supporting the robustness of these three biomarkers as key indicators of alveolar bone resorption.

### 3.6 Predictive performance of multiple linear regression models

**Figure 8:**
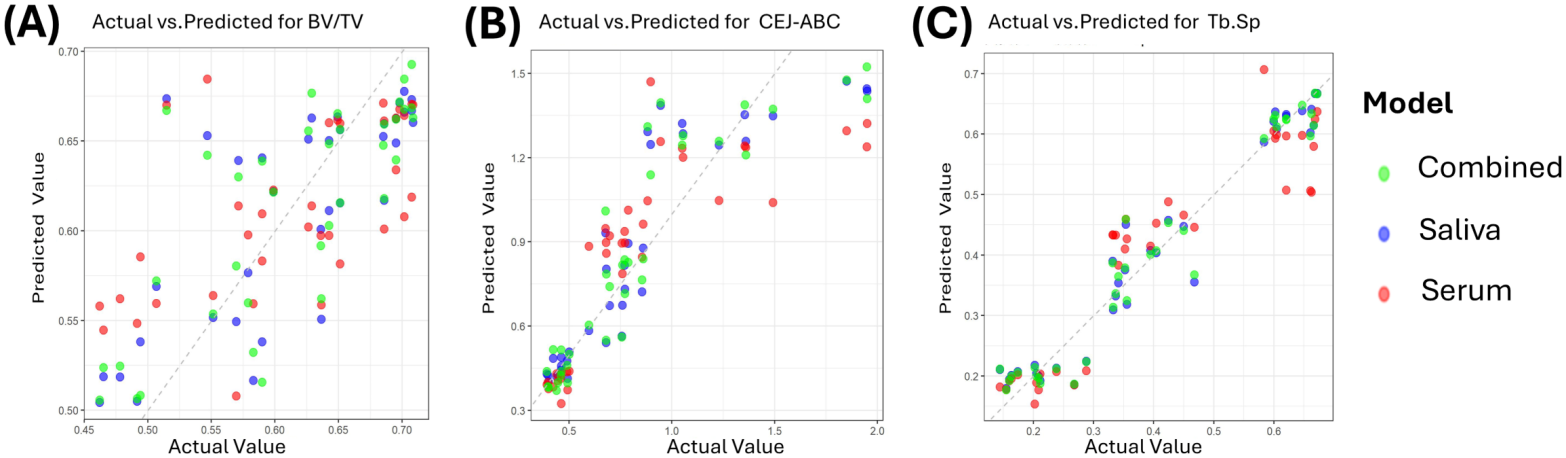
Scatter plots of predicted versus observed values and evaluation of regression model performance **(A)** BV/TV. **(B)** CEJ–ABC. **(C)** Tb.Sp.

Results from the three regression models (salivary factors, serum factors, and combined factors) were consistent with the correlation analyses. For BV/TV, the explanatory power of the models remained limited, with R^2^ values of only 0.56 and 0.36 for the salivary and serum models, respectively. Salivary factors demonstrated strong explanatory power for Tb.Sp and CEJ–ABC (R^2^ values of 0.95 and 0.78), outperforming the serum-factor models (0.86 and 0.64). After integrating the biomarkers, the overall predictive performance improved: MSE decreased and R^2^ increased for all target variables, with a significant improvement observed for BV/TV and modest gains for Tb.Sp and CEJ–ABC (**Figure 8**, **Table 1**).

**Table 1:** Performance metrics for predicting bone parameters using salivary, serum, and combined models.

Model name	Prediction objective	MSE	R^2^
Saliva Model	BV/TV	0.003	0.560
Serum Model	BV/TV	0.004	0.356
Combined Model	BV/TV	0.003	0.580
Saliva Model	Tb.Sp	0.002	0.951
Serum Model	Tb.Sp	0.005	0.856
Combined Model	Tb.Sp	0.002	0.952
Saliva Model	CEJ-ABC	0.004	0.778
Serum Model	CEJ-ABC	0.007	0.639
Combined Model	CEJ-ABC	0.004	0.790

The predictive performance differed markedly between the three-factor combination model (Top3) and the single-factor model (Top1). For BV/TV, all models yielded R^2^< 0.6, and the introduction of two additional factors failed to improve prediction, suggesting that BV/TV variation may be driven more by biomechanical factors not captured in the training dataset. For Tb.Sp, the Top3 model in both saliva and serum showed a substantial improvement over Top1, with R^2^ increased by 14.7% and 10.1%; Similarly, for CEJ–ABC, the Top3 models outperformed the Top1 models, with R^2^ gains of 9.4% and 7.9% (**Table 2**).

**Table 2:** Performance metrics for predicting bone parameters using the Top3 biomarker combination versus the Top1 single biomarker.

Model name	Prediction objective	MSE	R^2^
Saliva_top3	BV/TV	0.003	0.560
Saliva_top1	BV/TV	0.003	0.550
Serum_top3	BV/TV	0.004	0.356
Serum_top1	BV/TV	0.004	0.276
Saliva_top3	Tb.Sp	0.012	0.951
Saliva_top1	Tb.Sp	0.042	0.804
Serum_top3	Tb.Sp	0.068	0.856
Serum_top1	Tb.Sp	0.083	0.755
Saliva_top3	CEJ-ABC	0.002	0.778
Saliva_top1	CEJ-ABC	0.003	0.684
Serum_top3	CEJ-ABC	0.005	0.639
Serum_top1	CEJ-ABC	0.008	0.560

### 3.7 Immunohistochemistry

**Figure 9:**
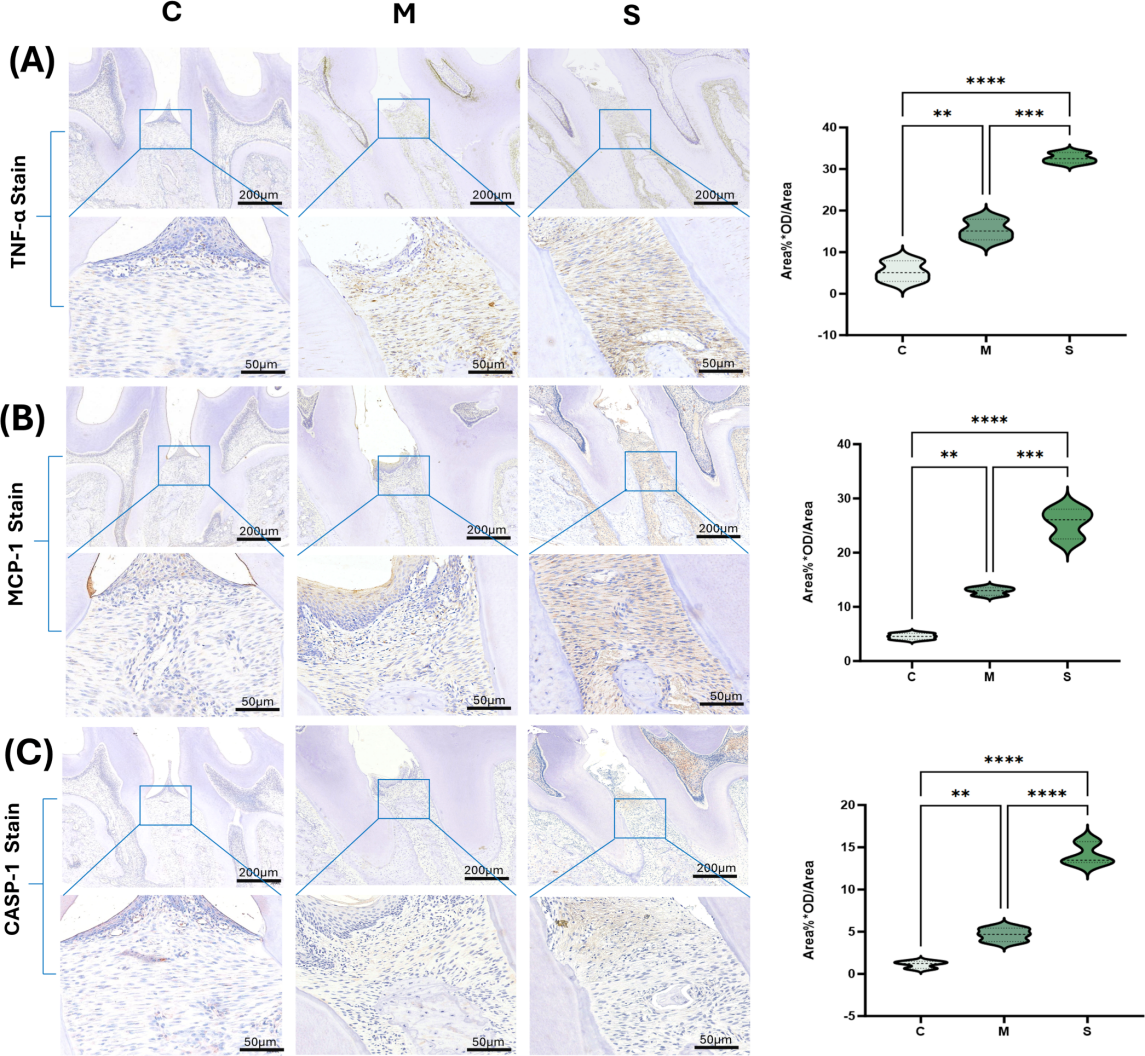
Immunohistochemical staining of key biomarkers in periodontal tissues **(A–C)** Representative images of TNF-α/MCP-1/CASP-1 staining in periodontal tissues (4× and 20×) and quantitative analysis of expression differences. *P < 0.05; **P < 0.01; ***P < 0.001; ****P < 0.0001.

Immunohistochemical staining showed that, compared with the C group, protein expression levels of MCP-1, TNF-α, and CASP-1 were significantly increased in the periodontal ligament, gingival epithelium, and infiltrated areas of connective tissue in the M and S groups (**Figures 9A–C**). The S group exhibited the strongest positive signals and the widest distribution within inflammatory infiltrates, and the differences were statistically significant (P < 0.05). These findings were consistent with the trends in biomarker concentrations detected in biofluids by the Luminex assay.

### 3.8 Multiplex immunofluorescence co-staining

**Figure 10:**
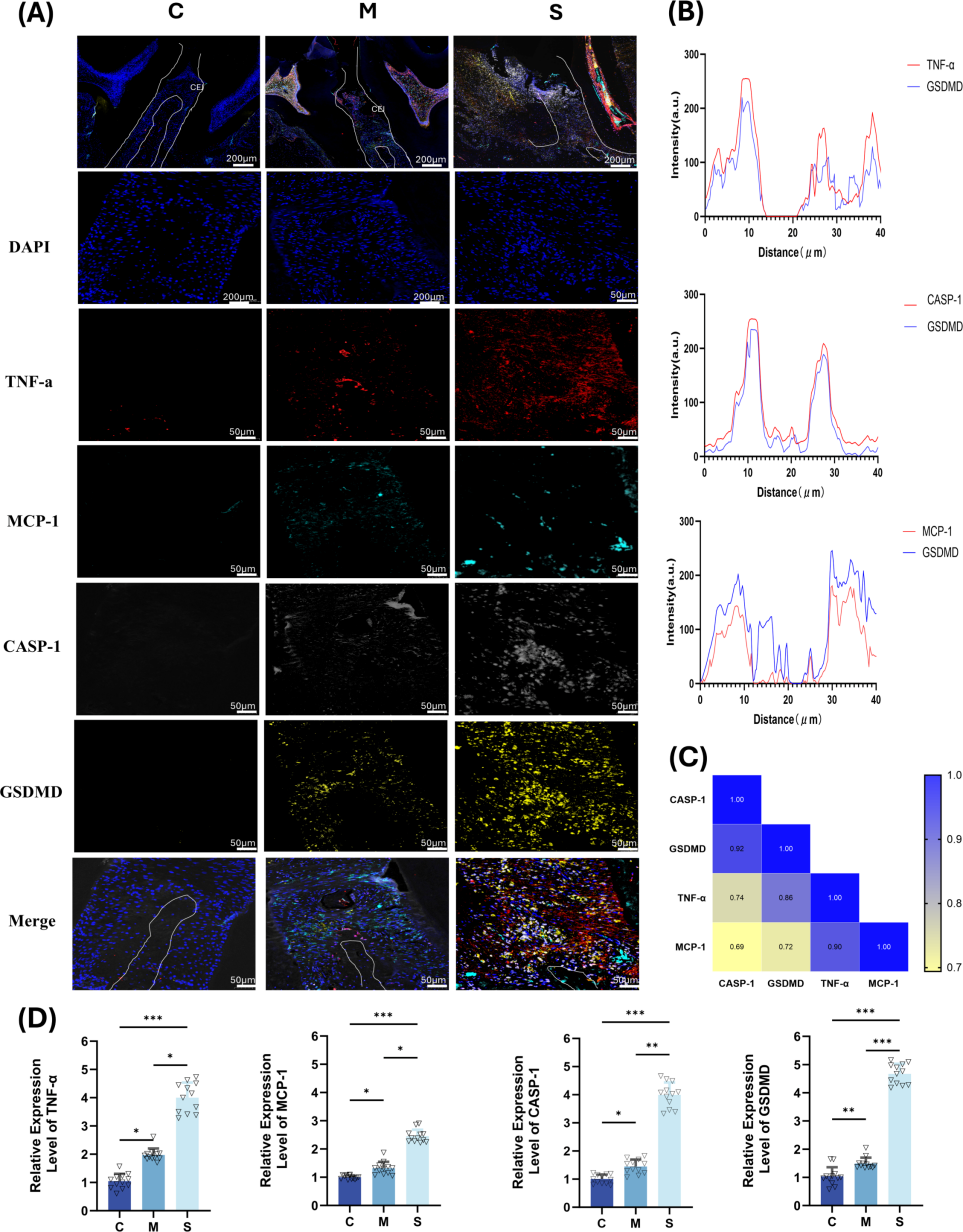
Immunofluorescence co-staining of key biomarkers and GSDMD in periodontal tissues **(A)** Representative fluorescence images from different groups. **(B)** Quantitative analysis of colocalization between key biomarkers and GSDMD. **(C)** Correlation heatmap of expression levels between key biomarkers and GSDMD. **(D)** Relative expression of biomarker fluorescence grayscale intensity compared with the control group.

Representative multiplex immunofluorescence images and quantitative analyses are shown in **Figures 10A–C**. Analysis of the spatial relationship and correlation between the key biomarkers and the pyroptosis execution molecule GSDMD showed that the expression levels of all four markers in the S group were significantly higher than those in the M group, accompanied by a clear shift in their distribution: in the M group, signals were mainly localized to the gingival epithelium, whereas in the S group, they extended to the periodontal ligament and surrounding inflammatory infiltrates. In infiltrated areas of the periodontal ligament and gingival tissues in the S group, MCP-1, TNF-α, and CASP-1 exhibited a high degree of colocalization with GSDMD. Quantitative analysis showed that the correlation coefficients between the signal intensities of these three biomarkers and GSDMD were high (R > 0.7, P < 0.05) (**Figures 10B, C**). These findings suggest that MCP-1, TNF-α, and CASP-1 are spatially associated with GSDMD-positive regions in advanced periodontal lesions and may be involved in pyroptosis-related inflammatory tissue injury during periodontitis progression. However, further functional studies are required to determine whether these biomarkers directly mediate pathological alveolar bone resorption and systemic inflammatory effects.

## 4 Discussion

With the continued development of point-of-care testing (POCT) technologies for periodontal biomarkers, increasing attention has been directed toward non-invasive and clinically translatable molecular tools. Öztürk et al. (2021) showed that active MMP-8 has value in the early diagnosis and staging of periodontitis, while Steigmann et al. (2020) highlighted the promise of multi-marker integration in biosensor-based diagnostic platforms. In addition, recent studies have explored salivary and serum inflammatory biomarkers in relation to periodontal status and disease progression (Alamri et al., 2023; Teles et al., 2024). However, most previous work has emphasized disease discrimination or treatment monitoring, whereas fewer studies have directly linked biomarker signatures to graded alveolar bone microstructural destruction. Therefore, the novelty of the present study lies not in proposing TNF-α, MCP-1, or CASP-1 as previously unknown periodontal molecules, but in integrating saliva and serum biomarker profiles with micro-CT-defined bone loss severity and machine-learning-based feature prioritization. This design provides a more structure-oriented framework for identifying biomarker combinations associated with active periodontal bone destruction.

Our results showed that, among the 21 biomarkers tested, inflammation-related factors contributed most prominently, and TNF-α, MCP-1, and CASP-1 ranked among the top contributors in both serum and saliva. The high contribution of TNF-α may be closely related to its role in enhancing osteoclast recruitment and activity and promoting the transition from local inflammation to bone resorption, potentially through activation of the RANKL*/*NF-κB signaling axis (Marahleh et al., 2019). Nevertheless, because RANKL and OPG were not directly measured in the present study, the involvement of this osteoclast-regulatory axis remains inferential and should be interpreted with caution. The high weight of MCP-1 may derive from its key chemotactic function (Nisha et al., 2018; Singh et al., 2021): by activating the CCR2 receptor, MCP-1 induces chemotaxis and infiltration of monocytes/macrophages into lesion sites, thereby providing an essential reservoir of precursor cells for sustained osteoclast differentiation.

Mahmood et al. (2023) reported that salivary CASP-1 shows high diagnostic sensitivity in patients with periodontitis. Compared with the terminal effector molecule IL-1β, CASP-1 may more sensitively reflect subclinical inflammatory status after treatment and thereby indicate potential risk of bone destruction (Tayman and Kızılgün, 2025), which is consistent with our finding that CASP-1 ranked among the key biomarkers. Mechanistically, CASP-1 may promote the processing and secretion of IL-1β, thereby initiating osteoclast differentiation and inducing alveolar bone resorption (Xu et al., 2022; Kim and Kim, 2020). Metabolic signals released from pyroptotic macrophages in local tissues may induce secondary pyroptosis in periodontal ligament stem cells, further suppressing their osteogenic differentiation and regenerative capacity and forming a vicious cycle of bone destruction (Sun et al., 2024). Accordingly, CASP-1 appears to play a critical role in inflammatory amplification and the progression of bone resorption in periodontitis, and effective regulation of CASP-1 may help inhibit bone destruction and promote bone repair after non-surgical therapy. Using multiplex immunofluorescence, we further examined the spatial distribution and correlations between the key biomarkers and pyroptosis-related proteins. TNF-α, MCP-1, and CASP-1 showed significant colocalization with the pyroptosis execution protein GSDMD within periodontal lesions (P < 0.05), mainly accumulating in areas with dense inflammatory infiltration and active osteoclastic activity. These findings suggest that the observed changes are not isolated concentration fluctuations; rather, these molecules may be linked to pyroptosis-related inflammatory responses within periodontal lesions. Nevertheless, the current evidence is based mainly on association and colocalization, and further functional validation is needed before a causal pyroptosis-mediated bone destruction pathway can be established. This is in line with the regulatory framework proposed by Shinohara et al (Shinohara et al., 2023; Kabacaoğlu and Öztürk Özener, 2024), in which the three factors act sequentially in inflammation initiation, inflammasome activation, and pyroptosis amplification to drive bone resorption. Notably, although IL-1β and MMP-8 showed stronger linear correlations (R) with micro-CT bone parameters, their feature-importance contributions for predicting graded alveolar bone resorption were lower than those of TNF-α, MCP-1, and CASP-1. This differs from some previous studies that emphasized IL-1β or MMP-8 as core biomarkers for periodontitis (Kim et al., 2020; Alarcón-Sánchez et al., 2025), suggesting differences in functional hierarchy among molecules during the bone destruction process. MCP-1–mediated monocyte chemotaxis and TNF-α–triggered inflammatory cascades represent earlier events, whereas CASP-1, as a key protease in inflammasome activation, serves as a hub that processes upstream inflammatory signals into bioactive IL-1β, thereby amplifying local inflammation and promoting pyroptosis-associated bone resorption. Previous cross-sectional screening has often focused on the presence or absence of lesions, tending to identify downstream effectors reflecting end-stage tissue destruction. In contrast, by establishing a micro-CT–based grading system to differentiate degrees of severity, our model is more sensitive to upstream signals that drive the progression of bone resorption. In clinical cross-sectional studies where quiescent samples constitute a relatively high proportion, biomarkers may present stable inflammatory signatures, thereby highlighting cumulative indices that reflect end-stage tissue destruction. Moreover, XGBoost is well suited to capturing nonlinear contributions and complex interactions among variables, preferentially retaining regulatory nodes with incremental explanatory value (Li et al., 2022), whereas traditional statistical tests primarily reflect linear correspondences between variables and bone parameters (Lundberg et al., 2020) and may underestimate hub-like regulators under certain conditions. However, given the relatively small sample size of the present study, these feature-importance rankings should be interpreted as exploratory rather than definitive. Additional explainability and stability analyses, such as SHAP values or permutation importance, would further strengthen the interpretability of the ranking results in future validation studies. Therefore, these biomarker sets may be complementary in diagnostic utility: IL-1β and MMP-8 may better reflect established tissue destruction, while TNF-α, MCP-1, and CASP-1 may better indicate whether bone resorption is in an active, progressive phase, providing a more forward-looking biological basis for identifying critical therapeutic windows in periodontal intervention.

In this study, bone microstructural parameters reflecting short-term bone remodeling activity were selected for quantitative correlation analysis with biofluid biomarkers. CEJ–ABC and Tb.Sp were more sensitive to early fluctuations in bone structure, showing significant associations with relevant biomarkers and demonstrating predictive performance, consistent with the findings of Fu et al (Fu et al., 2025). These parameters reflect short-term fluctuations in the balance between bone resorption and formation and represent intuitive morphological manifestations of accelerated remodeling (Kızıldağ et al., 2025), making them more likely to change in parallel with biofluid biomarkers such as MCP-1 and CASP-1. In contrast, BV/TV mainly reflects long-term cumulative bone mass and showed weaker associations with short-term biochemical fluctuations, consistent with reports by Wang et al (Wang et al., 2025). Combining biochemical biomarkers with dynamic structural parameters such as CEJ–ABC and Tb.Sp enables coordinated evaluation of alveolar bone destruction from both molecular and morphological perspectives, providing a basis for dynamic clinical monitoring.

Feature-importance analysis indicated that the contributions of TNF-α, MCP-1, and CASP-1 in saliva were relatively balanced, whereas MCP-1 was strongly dominant in serum. This suggests that saliva and serum have distinct information structures: saliva more closely reflects local microenvironmental and tissue metabolic changes, whereas serum more strongly represents stable signals within systemic inflammatory pathways. Traditionally, salivary samples have been considered to have limitations in clinical use, including relatively poor stability, lower biomarker concentrations, and more pronounced matrix interference (Erta et al., 2025). However, other studies suggest that these factors have limited impact on the discriminatory performance for periodontal status (Rocha et al., 2024). In our study, saliva showed a clearer advantage for detecting indicators closely related to tissue metabolism and bone resorption, such as CTSK and MMP-8, consistent with findings reported by Alamri and Teles (Alamri et al., 2023; Teles et al., 2024). Accordingly, different biofluids may be preferred under different application scenarios: serum may be more interpretable for assessing periodontal risk under a systemic inflammatory background or overall immune status, whereas saliva may more sensitively reflect local microenvironmental changes for staging local bone resorption or tracking lesion progression.

Changes in key serum biomarkers indicate a possible link between local periodontal tissue destruction and systemic immune imbalance. Chronic periodontal infection may increase the circulation of pro-inflammatory mediators, including TNF-α and MCP-1, which are commonly associated with sustained inflammatory responses. This pattern is also related to CASP-1-mediated pyroptosis, a pathway reported in other chronic inflammatory diseases such as diabetic nephropathy and atherosclerosis (Wen et al., 2022; Shi et al., 2025). Therefore, elevated serum CASP-1 levels may reflect not only local alveolar bone damage but also a broader inflammatory state. These overlapping features suggest that serum biomarkers could help monitor periodontal disease activity while also providing insight into systemic inflammatory conditions. However, classical systemic inflammatory markers such as C-reactive protein (CRP), which is widely used to reflect systemic inflammatory burden, were not included in the present biomarker panel. Future studies should incorporate CRP together with the current host-derived biomarker set to compare their relative value in capturing systemic inflammation versus active periodontal bone destruction.

Several limitations should be noted. Systemic inflammatory status was inferred from circulating biomarkers alone. Additional functional assessments, such as immune cell profiling or organ-level analysis, would provide a more complete picture of the systemic response linked to periodontal destruction. Another important limitation is that oral microbiome assessment was not included in the present study. This is particularly relevant in a ligature-induced model, in which microbial dysbiosis is a major upstream driver of periodontal inflammation and tissue destruction. In addition, the longer ligature duration used to model severe periodontitis may have introduced non-specific mechanical irritation or secondary inflammatory effects beyond progressive periodontal destruction itself, which could have influenced biomarker expression and mechanistic interpretation. RANKL and OPG were also not measured in the present study. Although TRAP staining and micro-CT supported the presence of osteoclast-associated bone resorption, the absence of these canonical bone remodeling regulators limited direct mechanistic evaluation of osteoclastogenic signaling and remodeling imbalance. Moreover, only male rats were included in this study. Although this design helped reduce hormonal variability in the initial experimental setting, it introduced sex bias and limited the generalizability of the findings, particularly because estrogen can influence inflammatory responses, osteoclast activity, and bone remodeling. In addition, the relatively small sample size and the lack of external validation may have affected the stability and generalizability of the XGBoost-derived feature rankings despite internal cross-validation. Although a prespecified outlier criterion was used during data preprocessing, detailed reporting of excluded values and formal sensitivity analyses with and without outlier exclusion were not performed in the current study, which may limit full assessment of result robustness. Therefore, although our data support associations between host-derived biofluid biomarkers, pyroptosis-related signals, and alveolar bone microstructural deterioration, they do not allow direct evaluation of host–microbe interactions or full characterization of bone remodeling pathways during disease progression. Mechanistically, current colocalization findings mainly provide spatial association clues and are insufficient to support causal inference among inflammatory responses, pyroptosis, and disordered bone metabolism. Future studies should incorporate microbiome profiling, finer time-point designs, RANKL/OPG-related remodeling markers, functional assays such as caspase-1 activity measurement and GSDMD cleavage detection, sex-balanced cohorts, and external validation in independent animal and human populations to better define the upstream and downstream mechanisms of periodontal bone loss.

## 5 Conclusions

This study proposed and validated an interpretable association between key biofluid molecules and the severity of bone destruction. Key biomarkers, including TNF-α, MCP-1, and CASP-1, were associated with the severity of alveolar bone destruction and showed potential links to pyroptosis-related tissue injury. These biomarkers may also be relevant to the progression of periodontitis-associated systemic inflammatory changes, although direct functional validation is still required. These biomarkers show potential value for grading bone resorption and for screening targets for non-surgical therapy, while also providing a basis for candidate target selection and model development for integrated multi-analyte detection.

## Data Availability

The original contributions presented in the study are included in the article/**Supplementary Material**. Further inquiries can be directed to the corresponding authors.
