# Supplementary material for: The inflammation-pyroptosis axis links local and systemic biomarker profiles to active alveolar bone microstructural deterioration
Source: Front Physiol. 2026 May 13;17:1814322. doi: 10.3389/fphys.2026.1814322 (PMC13212031; doi:10.3389/fphys.2026.1814322)
Supplement: Supplementary file 1 [file SupplementaryFile1.docx]

Supplementary Material

## Supplementary Figures


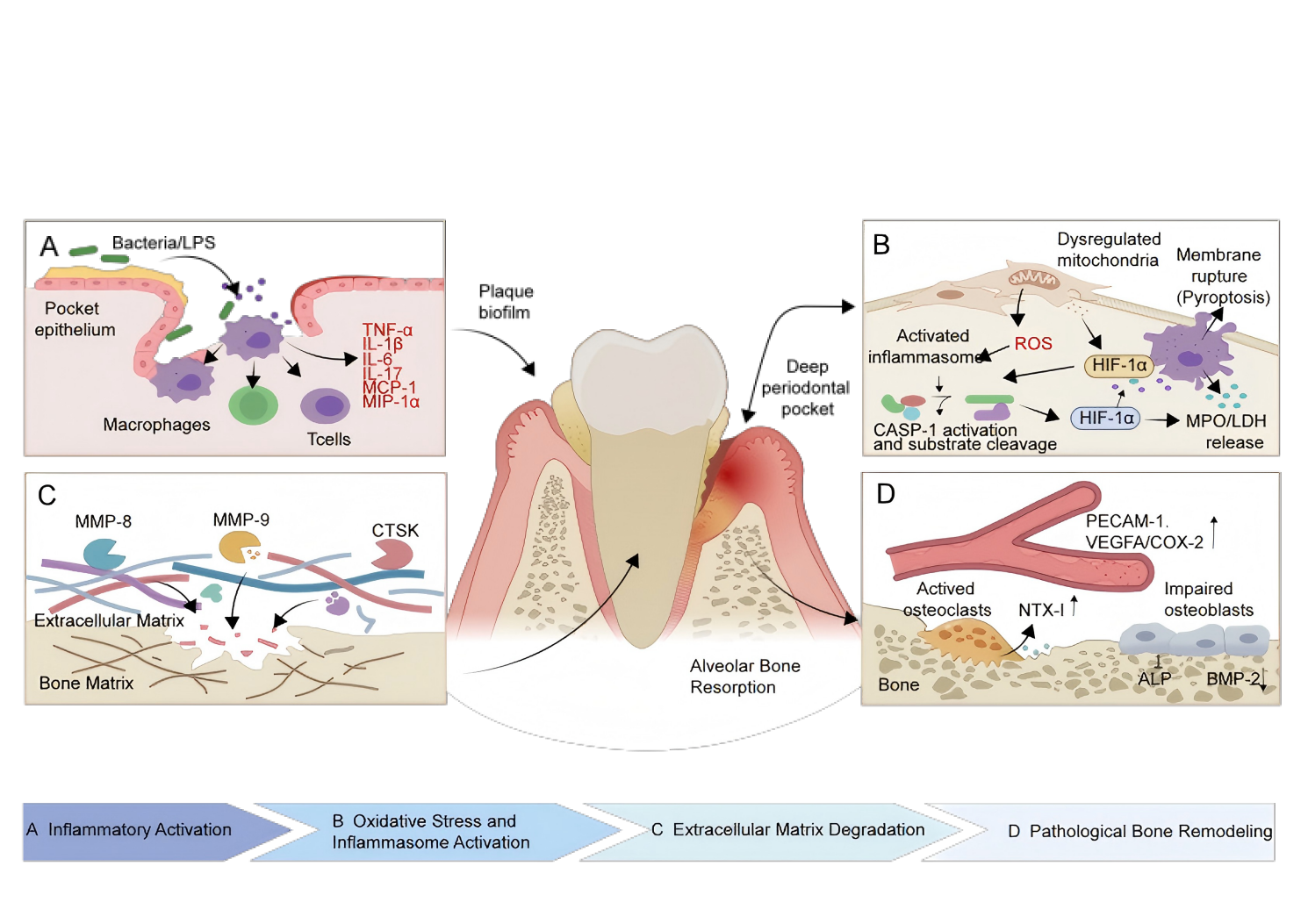


**Supplementary Figure 1.** Overview of the Classification of Biomarkers Associated with Alveolar Bone Resorption in PeriodontitisSchematic diagram showing that periodontal pathogens trigger inflammatory cascades, oxidative stress, and matrix degradation, and that dysregulated tissue remodeling and bone metabolism ultimately lead to alveolar bone resorption.

Publication License：<https://BioRender.com/m0nh909>
